# Navigating menstrual stigma and norms: a qualitative study on young people’s menstrual experiences and strategies for improving menstrual health

**DOI:** 10.1186/s12889-024-20936-5

**Published:** 2024-12-17

**Authors:** Eva Åkerman, Anna Wängborg, Maria Persson, Renita Sörensdotter, Marie Klingberg-Allvin

**Affiliations:** 1 Department of Women’s and Children’s Health, Karolinska Institutet, Tomtebodavägen 18A, Stockholm, 171 77 Sweden; 2 Department of Public Health Sciences, Stockholm University, Albanovägen 12, Stockholm, 106 91 Sweden; 3 Centre for Gender Research, Uppsala University, Villavägen 6, Uppsala, 752 36 Sweden

**Keywords:** Menstrual health, Stigma, Qualitative study, Healthcare, Mental health, Sexual health, Gender equality

## Abstract

**Background:**

Menstrual health is a recognised important public health issue and is essential for the realisation of gender equality and the achievement of Sustainable Development Goals. This study aimed to explore the menstrual health experiences of young people in Sweden and how the menstrual cycle affects their health and lives. The study also aimed to identify the facilitators and barriers to achieving menstrual health.

**Methods:**

We conducted a qualitative study in Sweden. Sixteen young people aged 18–28 who have experienced the menstrual cycle participated in individual interviews. Purposeful sampling combined with snowball sampling was applied to recruit the participants. The data were analysed using reflexive thematic analysis.

**Results:**

Participants viewed menstruation as a sign of having a healthy and functioning body. Menstruation was linked to becoming a woman and fostered a sense of community and sisterhood, which was viewed as positive among cis women. Further, the results showed that physical and emotional symptoms related to the menstrual cycle limited the participants’ everyday lives and social relationships and had a negative effect on their sexual and mental health. While managing their emotional discomfort and other menstrual complaints, they also had to deal with the public stigma and norms about menstruation contributing to shame and worries. Barriers to menstrual health included stigma and norms related to menstruation, which led to the adoption of expected behaviours, such as avoiding participation in social activities. The normalisation of menstrual complaints also contributed to delays in seeking healthcare, despite having symptoms that had a negative effect on their health. An important factor promoting menstrual health and quality of life is access to prompt treatment to mitigate and decrease symptoms that limit everyday life.

**Conclusions:**

The results indicate that menstrual stigma and related norms create challenging situations limiting menstruating people’s everyday lives and reluctance to seek healthcare despite needing to. To promote the menstrual health of menstruating young people in Sweden, organised and systematic screening of menstrual cycle-related symptoms should be provided within student health services at schools and universities, and primary healthcare. Policymakers should consider integrating stigma-reducing efforts into public health interventions to improve general awareness and promote gender equality.

**Supplementary Information:**

The online version contains supplementary material available at 10.1186/s12889-024-20936-5.

## Background

Menstrual health is a recognised important public health issue and is essential for the realisation of gender equality and the achievement of the Sustainable Development Goals [1, 2]. Menstrual health is defined as ‘a state of complete physical, mental, and social well-being and not merely the absence of disease or infirmity, in relation to the menstrual cycle’ [3]. Achieving menstrual health involves five essential requirements: (i) access to accurate, timely and age-appropriate information about the menstrual cycle throughout the lifespan; (ii) access to affordable menstrual products, supportive facilities and, water and sanitation hygiene services, for changing menstrual products and cleaning and/or disposing of used materials (iii) access to timely diagnosis, treatment, and care for menstrual cycle-related discomforts and disorders, including health services and resources such as pain relief and strategies for self-care; (iv) the ability to experience a supportive social environment free from stigma and psychological distress; and (v) the freedom to participate fully in all aspects of life without menstrual-related exclusion, restrictions, or discrimination [2, 4]. The requirements highlight that menstrual health is a human rights issue, as it ensures access to health, dignity, and equality, allowing everyone who menstruates to participate fully in society without stigma or discrimination. Further, the definition acknowledges that not all people who menstruate identify as women or girls. Others who menstruate may be transgender men or non-binary individuals. In this study, the term ‘menstruating young people’ encompasses all individuals who menstruate, including girls, women, transgender, and non-binary individuals from diverse social backgrounds.

Menstrual health affects half of the world’s population for up to 40 years of their lives. However, menstrual health has historically been under-researched and a neglected public health issue. In high-income countries, there have been increasing efforts to understand and address menstrual disorders, such as dysmenorrhea, endometriosis, heavy menstrual bleeding and premenstrual syndrome (PMS), and to evaluate the treatment of menstrual disorders [5–9]. Dysmenorrhea, also known as menstrual pain, is the most frequent menstrual disorder and affects approximately 70–90% of young women worldwide [10]. PMS is characterised by recurrent physical and emotional symptoms one to two weeks before the start of each menstrual period and is reported by approximately 30–40% of women of reproductive age [11]. Heavy menstrual bleeding affects at least a quarter of reproductive-age menstruators worldwide [12]. Menstrual disorders can negatively affect the health and quality of life of menstruating people and limit their ability to participate in activities, work and education [6, 13–20]. Hence, menstrual-related symptoms can exacerbate gender inequalities, affecting economic status and social participation, which is why menstruation is increasingly recognised as a matter of gender equality and public health concern [21].Furthermore, research shows that the normalisation of menstrual pain and heavy bleeding means that those affected are less likely to seek care, particularly young people [22, 23]. Another factor that affects care seeking among menstruating people is menstrual health literacy—that is, the ability to obtain access to, understand, value and use information on menstrual health [22, 23]. An increasing number of studies have shown that young menstruating people have insufficient knowledge about menstruation [22, 24] and, therefore, have difficulty identifying related symptoms [23]. Mothers and healthcare personnel are considered crucial in conveying knowledge and information [25–28]. Evidence has demonstrated that mothers play a crucial role as a source of information regarding menstruation, particularly for adolescents navigating their experiences of menarche [29]. Furthermore, a systematic overview of experiences of menstruation, encompassing 104 studies from high-income countries, showed that menstruation was stigmatised in more than half of these countries [17].

In Sweden, a recent study among adolescent schoolgirls reported that 93% had menstrual symptoms with 81% experiencing at least one moderate symptom and 31% reporting at least one severe symptom [30]. The most frequent symptoms were dysmenorrhea (80%) and mood disturbance (81%), followed by irregular periods (68%), heavy bleeding (60%), and other general symptoms (60%). Another Swedish study among adolescents reported that 14% had monthly absence from school because of dysmenorrhea [31]. Despite the widespread menstrual symptoms among young people, qualitative research addressing menstrual health experiences, which includes both negative and positive aspects among young people in Sweden is limited. Exploring menstrual health experiences in young people is critical, as insight into their lived experiences can serve as a knowledge base for early interventions that promote physical, mental, and social well-being.

From a public health perspective, understanding the barriers and enablers of menstrual health is crucial for creating inclusive public health policies that promote well-being. Therefore, this study aimed to explore menstrual experiences and how the menstrual cycle affects the health and well-being of menstruating young adults in Sweden. It also aimed to identify the facilitators and barriers to achieving menstrual health.

### Theoretical framework

Theories of gender norms and menstrual stigmatisation permeated the analysis and discussions in this study.

### Gender norms and menstruation

How menstruation is experienced and interpreted is affected by cultural norms, values and biological processes. Cultural norms for gender and the body are central to understanding menstruation. Gender norms determine how people are expected to act and live their bodies depending on their assigned sex [32]. Menstruation is a symbol of womanhood, and therefore it is associated with certain behaviours and certain ways of interpreting one’s body [33]. Norms for gender and menstruation create assumptions and expectations of how menstruating people are and should be [33, 34], for example, that only women menstruate and that all women menstruate [34]. Menarche, the onset of menstruation, is the cultural starting point for becoming a heterosexual reproductive woman, regardless of whether one has another gender identity or no desire to have children [33]. In connection with becoming a woman, menstruation is managed differently depending on gender identity, sexual orientation and ethnicity.

### Menstrual stigma

In different societies and cultures, the menstrual blood is viewed as dirty and a taboo [33]. This contributes to the process of stigmatisation. Menstrual stigma entails a negative view of menstruation and of menstruating people, which is reinforced through norms and values [35]. According to Johnston-Robledo et al., menstruation can be viewed as an invisible social stigma for women [36]. Menstrual blood becomes a stigmatising attribute, as it is interpreted as dirty and unclean and as something that menstruating women should conceal [17]. The social stigma around menstrual blood can lead women who initially saw menstruation positively—as a natural part of womanhood—to adopt restrictive behaviours of secrecy to avoid negative judgment [37]. Menstruating women are also stereotypically viewed as irrational, hysterical and emotionally unstable [38] and different from non-menstruating women or men, which contributes to stigmatisation [36]. Menstrual stigma is attributed to menstruating women, but this conceals the fact that it also affects menstruating people who do not identify as women [36]. McHugh’s introduce the term “menstrual moaning”, which refers to women’s negative communication about menstruation. McHugh suggests that menstrual moaning, by reinforcing negative cultural views of women’s bodies as flawed, deficient, and diseased, can harm women’s attitudes toward menstruation and perpetuate menstrual shame [39].

## Methods

### Study design

We conducted an exploratory qualitative study based on in-depth interviews with young people aged 18–28 years with experience of menstruation. The exploratory and in-depth approach allows for an open investigation into the lived experiences of menstrual health. Qualitative study designs are appropriate for exploring people’s perceptions and experiences, providing deep insights into their thoughts and underlying factors that can inform future health-promoting efforts [40].

### Recruitment

Eligible participants were young adults aged 18–29 years with experience of the menstrual cycle and preferably without any previous menstruation-related diagnosis. Purposive sampling was used to recruit participants with various backgrounds regarding age, education background, gender identity, country of birth and geographic location (rural or urban setting) [41]. Snowball sampling was used to recruit ‘hard-to-reach participants’, such as participants with foreign-born backgrounds and migrant experiences [42]. Recruitment was conducted outside healthcare centers to better reach individuals without menstruation-related diagnoses, as this was one of the eligibility criteria.

An information letter about the study was sent to several universities, non-governmental organisations, Swedish language schools for immigrants and meeting places organised by municipalities. The recipients were asked to post the information letter in physical facilities and disseminate it to potential participants. The information letter contained contact details to the research team, which interested participants were instructed to use to register their interests or if they had any questions about the study.

To reach menstruating people with trans experience, an ad specifically aimed at trans men was created on the Karolinska Institute website. Non-governmental organisations for lesbian, gay, bisexual, transgender and queer (LGBTQ) people were asked to disseminate the ad. This resulted in a few transmen contacting the research group and participating in interviews.

### Data collection

A semi-structured interview guide was created, pilot tested and revised covering the following topics: experience of menarche; support and knowledge of menstruation during adolescence; current experience of menstruation and the menstrual cycle; the effect of menstruation and the menstrual cycle on physical, mental and sexual health; effect on everyday life and living habits; and knowledge and sources of information on and attitudes towards menstruation (see Additional file 1). These topics were chosen to provide a comprehensive understanding of how menstrual cycle-related symptoms impact individuals across different stages of life and areas of health.

In total, 16 individual interviews were performed from July 2022 to January 2023. Saturation was reached after 16 interviews with regard to participants’ experiences of common menstrual cycle-related symptoms and menstrual stigma, meaning no additional insights emerged in the later interviews within these domains. The sample size of 16 interviews was deemed sufficient for this qualitative study, as the data were rich and provided a robust range of insights to thoroughly address the research questions in depth. The participants could choose whether to be interviewed digitally through Teams or through physical meetings. In total, 15 interviews were held through Teams and one over the phone. The interviews lasted for 45–95 min and were digitally recorded and transcribed verbatim. The quotations underwent minor language editing for readability without changing their meaning.

### Participants

The interview participants were 18–28 years old, with an average age of 24 years (Table 1). Most were born in Sweden, one was born in another Nordic country, and two were born outside Europe. The majority identified as women, with two identifying as men. Half of the participants had a partner, and a third were single. The majority were studying and/or working. All participants had finished secondary school, and more than half had chosen to study at an institution of higher learning. The participants lived in six different regions, including both urban and rural areas.

**Table 1:** Participants’ characteristics

**Age** Year	**Range (Mean)** 18–28 (24)
**Gender identity** Woman Man	**n** 14 2
**Country of birth** Sweden* Nordic country Sub-Saharan Africa	**n** 13 1 2
**Immigration year** 2015–2020 2005–2010	**n** 1 2
**Education level** More than 12 years University or higher education	**n** 11 5
**Occupation** Working Studying Other	**n** 9 6 2
**Marital status** Single Married/cohabiting Non-cohabiting partner Other	**n** 5 8 1 2
**Menarche** Year	**Range (Mean)** 10–14 (12.2)

*One with parents born in Sub-Saharan Africa

### Data analysis

The transcribed interviews underwent reflexive thematic analysis, as described by Braun and Clarke [43]. Thematic analysis aims to identify and present themes in participants’ stories. An abductive approach was used in the analysis—that is, a combination of inductive and deductive approaches. Five researchers (EÅ, AW, MP, RS and MKA) with different preunderstandings, competences and professions participated in the analysis process. The first step in the analysis process involved becoming familiar with the data collected, which means that the researchers listened to the audio files and read the transcribed interviews. In the initial phase of coding, the researchers (EÅ, MP) identified different kinds of codes (e.g. *value codes*,* emotion codes*,* in vivo codes*,* process codes*) that could be used to capture as much as possible the participants’ stories based on the study’s purpose and the research questions [44].

All the transcribed interviews were coded by at least two researchers (EÅ, MP) using the software programme NVivo, which was also applied in sorting and developing the themes. The codes were then discussed by MP and EÅ, and they were added and adjusted. During the fine-tuning of the codes, notes were taken, and discussions on the preliminary themes were conducted. In parallel with this phase, the so-called ‘memo’ was developed for each participant to capture the individual stories in brief. The codes and identified themes were continually discussed by the research team (EÅ, AW, MP, RS and MKA) and revised throughout the analysis process.

### Researcher characteristics and reflexivity

The interviews were conducted by two researchers (EÅ and AW), both of whom had previous experience conducting interviews. The research team consisted of women who identified as cis-gender and who were native Swedish (AW, MP, MKA), with one (EÅ) born in Thailand. EÅ has been living in Sweden since she was 10 years old; her father originated from Sweden, and her mother originated from Thailand. One of the researchers (RS) identified as a queer lesbian. The researchers work in a predominantly white academic environment. The entire team has research experience working with sexual and reproductive health and rights, including issues such as migration. EÅ is a public health specialist and postdoctoral researcher. AW is a midwife and a doctoral student. MP is a doctoral student with a background in political science. RS is an associate professor in gender studies. MKA is a midwife and professor of reproductive health. The fact that all researchers involved in the analysis have or have had experience with menstruation means that they have a preunderstanding of the topic.

### Ethics approval and consent to participate

Ethics approval was obtained from the Swedish Ethical Review Authority (registration number 2022-03122-01). Informed consent was obtained from all participants before their participation. They received both written and oral information about the purpose of the study before the interviews took place. The participants were informed that participation was voluntary and that they could withdraw from the study at any time without providing a reason. They were also informed that their answers would remain confidential and that identifying information would be deleted from the transcripts. All the participants agreed to have the interviews recorded. After completing the interview, participants were encouraged to contact the researcher if any questions arose or if they wished to seek healthcare support and required assistance in finding appropriate health services.

## Results

**Fig. 1:**
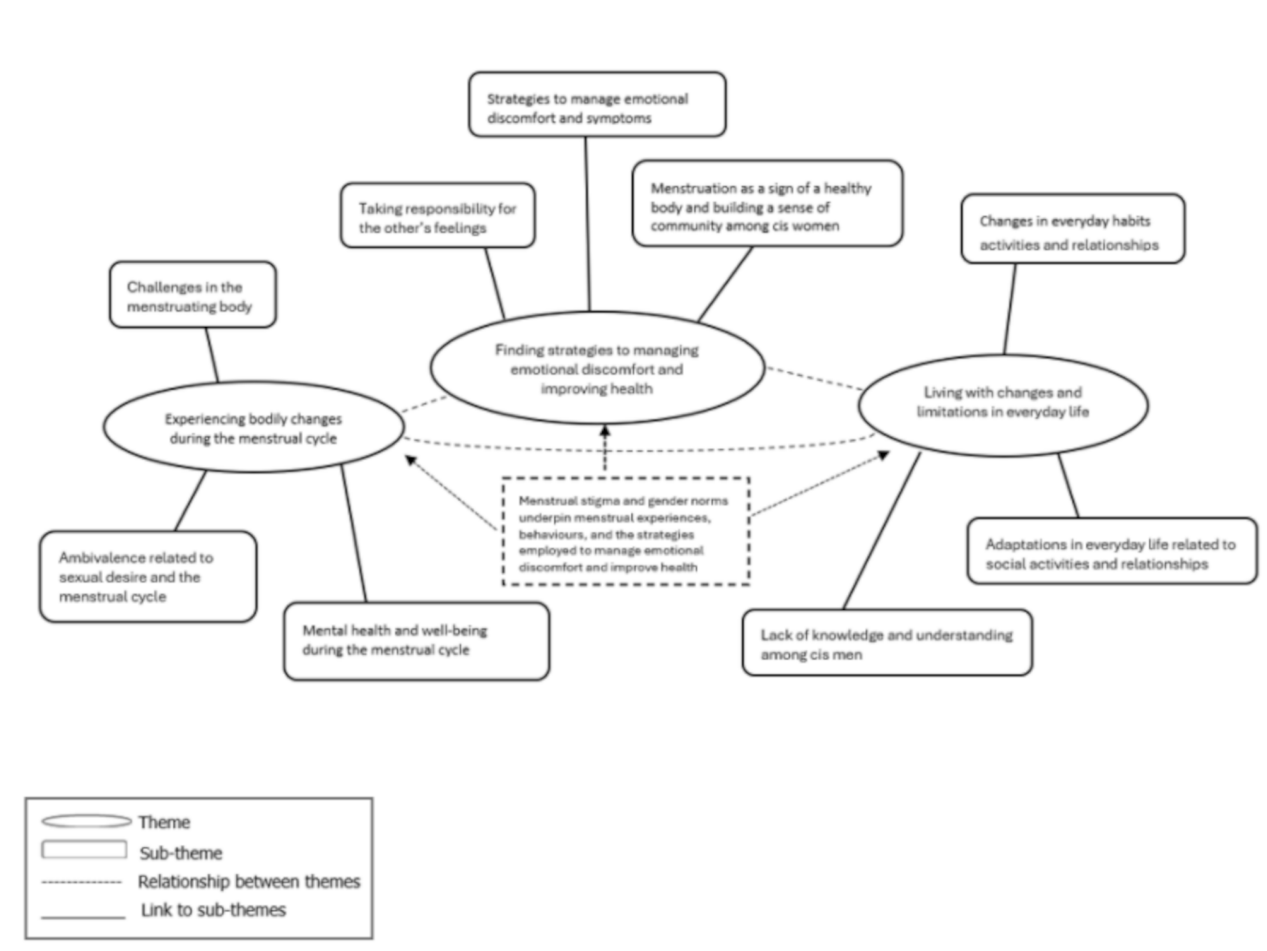
Themes and sub-themes

The analysis of the interviews resulted in three themes with related subthemes, which are presented in Fig. 1. In summary, results showed a nuanced picture of participants’ experiences of the menstrual cycle, ranging from viewing menstruation as a sign of having a healthy and functioning body to experiencing emotional and physical discomfort, highlighting the diversity of perspectives among participants. Further, Fig. 1 illustrates a relationship between the theme ‘Finding strategies managing emotional discomfort and improving health’ and the other themes. This illustrates, that regardless of the severity of menstrual symptoms, participants employed strategies to manage emotional discomfort, and those experiencing severe complaints adopted additional strategies to improve their health. The illustration also demonstrates how menstrual stigma and gender norms underpin menstrual experiences, behaviours, and the strategies employed to manage emotional discomfort and enhance health. These experiences further illustrate how participants navigate stigma and norms related to menstruation, finding ways to cope with menstrual stigma while managing their menstrual cycle-related symptoms within these societal constraints.

The participants’ pronouns used in the results were based on each participant’s self-identified gender. The names given for each quotation below are pseudonyms used to maintain confidentiality.

### Experiencing bodily changes during the menstrual cycle

This theme describes the participants’ experiences of the menstrual cycle and associated physical symptoms. It also relates to norms and stigma about menstruation and the participants’ views of menstruation and its effect on their sexual and mental health.

#### Challenges in the menstruating body

All the participants experienced, or had experienced, that their menstruation physically affected them. Some participants felt tired or drained during menstruation and described their bodies as bloated. Others experienced headaches or migraines, while others experienced fever. Menstrual pain was also common and could range from manageable to excruciating. To understand their own experiences, some participants compared them with the menstrual complaints experienced by others. This sometimes made their own complaints easier to bear. Isabelle, who had severe menstrual pain, expressed the following:

*I’ve had menstrual pain. I’ve also heard about people who have fainted or vomited and so on*,* but I’ve never been in that much pain. This could support what I was thinking—that maybe it wasn’t an abnormal pain*,* either. Because I wasn’t vomiting*,* so maybe it wasn’t*,* like*,* painful enough. (Isabelle*,* 24 years old)*

Those who experienced heavy bleeding and severe menstrual pain viewed menstruation as tiresome, time-consuming and something they simply had to endure. The participants’ experiences also revealed stigmatising beliefs towards menstruation. Some viewed menstruation as disgusting; for example, Isabelle described menstrual blood as ‘*a bodily fluid you don’t want to be in contact with*’. This also caused some participants to feel unclean during menstruation. For Andreas and Robin, who are transgender men, the menstrual challenges they faced were not limited to only menstrual complaints. Gender norms recognising menstruation as a feminine experience and womanhood made them experience gender dysphoria connected to menstruation: ‘*Menstruation is very connected to gender dysphoria for me’ (Andreas*,* 28 years old).*

Andreas described menstruation as a vicious monthly reminder and looked forward to eliminating it. Andreas did not use contraception to stop his menstruation because he heard that it could increase breast size. However, not menstruating was important for his gender identity. Robin used a birth control implant to avoid menstruation, which was important for his gender identity. Both Robin and Andreas were on the waiting list for assessment ahead of gender-affirming surgery.

#### Mental health and well-being during the menstrual cycle

The physical symptoms that the participants experienced during menstruation also affected their mental health. Denise, who experienced recurrent heavy menstrual bleeding each month, described how this affected her mental health:

*I have very low—this sounds so melodramatic but—zest for life. Usually*,* I’ll just spend a lot of time lying on the couch. Partly because I don’t have*,* like*,* the mental wherewithal but also because I find it more work to be out and about when I have my period. (Denise*,* 21 years old)*

Several participants described feeling depressed and having less zest for life during menstruation. ‘*I get high levels of anxiety and feel tension in my body. The entire situation is very uncomfortable due to pain’ (Julia*,* 25 years old).*

The participants also reported that their mental health and well-being were affected during the premenstrual phase. They called these symptoms PMS or mood changes. Feeling sad, irritable and angry and having negative thoughts about themselves were among the reported emotional discomforts.

*You get sad easily. I usually start crying as soon as someone says anything. That I can do any stress management. As soon as I’m stressed and starting my period*,* things can just explode—little things I usually don’t care about. (Farah*,* 26 years old)*

Andreas had depressive symptoms but was unsure if they were due to gender dysphoria or PMS. Others also had difficulty understanding where emotions, such as anger or sadness, came from. Some stated that it was hard to explain these emotions to others. When a connection was made between mental well-being and the menstrual cycle, this had a calming effect on them. ‘*Thinking that maybe this isn’t about me or that I’m depressed or truly sad*,* but that it’s my hormones right now*,* I can feel like… that’s an enormous relief’ (Hanna*,* 22 years old).*

#### Ambivalence related to sexual desire and the menstrual cycle

The participants’ narratives revealed examples of norms and menstrual stigma related to sexual practices. Some felt that increased sexual desire during menstruation was strange, causing mixed emotions. ‘*I could feel a bit disgusting*,* but I was also very horny*,* as I’ve said. So it was a kind of strange feeling’ (Kajsa*,* 24 years old).* Avoiding sexual activity was also related to menstrual stigma. Hanna felt that there was a taboo regarding sex and menstruation and had the experience that men did not want to have sex with her when she was menstruating.

*That’s something I’ve experienced during relationships with various guys— that you aren’t supposed to have sex when you’re menstruating. […] They don’t want to have sex when I’m menstruating. […] My partners have often said*,* like*,* well*,* for your sake*,* we shouldn’t be having sex while you’re menstruating. (Hanna*,* 22 years old)*

Norms and religion caused some participants to avoid sex during menstruation, even if they had increased sexual desire. Two of the participants reported that, in Islam, it is forbidden to have sex during menstruation. *‘For us… it doesn’t interfere with anything. I don’t know*,* it might be different for others. For example*,* I’m Muslim*,* and when I have my period*,* we don’t have sex’ (Mariam*,* 26).*

Some participants described having increased sexual desire during menstruation as positive. One of the participants perceived it exciting to have unprotected sex at the start of her menstrual cycle, which contributed to increased sexual desire.

*At the start of my period*,* I usually allow myself to have unprotected sex. So maybe it’s more about my libido on a psychological level*,* if that makes sense—knowing*,* like*,* okay*,* this is a risk*,* but I’ve been doing it this way for two years*,* and it’s worked out*,* so I choose to continue doing it this way. (Clara*,* 23 years old)*

The participants who had severe menstrual pain, heavy bleeding and tenderness during menstruation due to fungal infections or chafing from pads had no sexual desire. One of them was Denise, who had heavy bleeding and other symptoms related to menstruation. A lack of sexual desire caused her to doubt her feelings for her partner and her sexual orientation. Julia, who had irregular menstruation and heavy bleeding, felt concern ahead of each menstruation, which contributed to decreased sexual desire.

*Yeah*,* after my period*,* I usually feel better and*,* like*,* my body is lighter*,* and that’s probably when my sex drive is the highest. But right before it*,* that’s usually when I feel concerned. I don’t have that much of a sex drive because I’m afraid that it’ll start at any time*,* and then I’m more cautious. (Julia*,* 25 years old)*

Some participants experienced increased sexual desire immediately after menstruation or during ovulation. Ester described being sexually frustrated during ovulation. Andreas also felt that ovulation affected his sexual desire. Strangely, he found men attractive during ovulation, which was confusing to him because he was not usually attracted to men.

### Living with changes and limitations in everyday life

This theme describes how the menstrual cycle affects the participants’ everyday habits, such as exercise, diet, social activities and relationships. It also includes the participants’ behavioural adaptations in relation to their menstrual cycle and their experiences of not being understood by cis men.

#### Changes in everyday habits

With regard to physical exercise and dietary habits, most of the participants felt an effect during the days before and during menstruation. How the participants experienced or felt about exercising depended on where in the cycle they were and what type of symptoms they experienced. Menstrual stigma was also noticeable in the participants’ behaviour adoption, with efforts to hide their menstruation during physical exercise. Denise, who had heavy bleeding, avoided exercising during menstruation partly because she felt that one should not use a pad under training tights and partly because she could not use tampons due to vulvodynia.

*Menstruation means that I can skip exercising or that when I do exercise*,* I have to go to the bathroom all the time because I feel that I can’t wear a pad under these tight training bottoms. So I use regular pantyliners and I bleed through them right away. There have been several times when I’ve been at the gym despite my period and have had a total breakdown*,* gotten sad and had to go home. (Denise*,* 21 years old)*

Ester and Noor felt strong and could perform well during menstruation, contrary to the expectation of feeling weak. Noor, despite experiencing heightened energy levels, sometimes struggled with severe menstrual pain when attempting to exercise. Sofia described that she had problems with sore breasts when playing football. To participate in football practice, she needed to bind her breasts, which led to breathing difficulties. Now competing in another sport at the elite level, Sofia adjusted her exercise routine based on her menstrual cycle phase.

*You can perform at your maximum during the week of your period; you are at peak recovery capacity while you are bleeding. Then*,* the week after*,* the body is very weak*,* so you should focus more on recovery. Therefore*,* yeah*,* since I’m a woman and an elite athlete*,* that’s important information*,* as the body reacts differently at different phases of the menstrual cycle. (Sofia*,* 27 years old)*

Some participants also felt that it was more difficult to exercise during menstruation because they felt depressed or sad, it was physically uncomfortable or they felt bloated.

Other changes related to the menstrual cycle included increased hunger, with some eating more often and craving certain types of food, such as fast food or fatty food and sugar, particularly chocolate. Ester, a long-term vegetarian, described that she would crave liver pâté, and she interpreted it as a signal that her body needed iron and thus took iron supplements. For Farah, it was important to eat energy-rich food during menstruation, as the body was losing blood.

#### Adaptations in everyday life related to social activities and relationships

Everyday life was affected differently depending on the menstrual symptoms that the participants experienced. Those with heavy bleeding or severe menstrual pain often perceived menstruation as a limitation in everyday life and avoided social activities. Denise described living a different life during menstruation due to heavy bleeding. She avoided social activities outside her home because they involved much planning and effort to hide her menstruation, from choosing the right colour clothes to bringing extra clothing in case of leakage.

*Even if I want to wear light trousers or something*,* I have to wear black all the time*,* and I need to plan ahead if I’m doing anything in particular. I have to plan really far in advance to make sure I’ll manage*,* like what I’ll wear and what to bring along. Like*,* I always bring extra underwear and trousers everywhere*,* and there’s so much planning*,* a lot of planning and adaptation. (Denise*,* 21 years old)*

The same participant also avoided applying for work in workplaces where light work clothes were needed. Participants who did not have problems with heavy bleeding also stated that they chose clothing based on colour and size. Due to feeling bloated during menstruation, some participants preferred to wear larger underwear and trousers. Nina, who worked in healthcare and had to wear white scrubs, felt that it was a hassle having to think about where the bathrooms were located to always be ready to change menstrual pads or tampons and avoid leakages.

Participants with severe menstrual pain often stayed at home during menstruation because the pain made it impossible to get out of bed or stand up straight. Julia described her pain as very unpredictable, which caused her to avoid planning activities. Both Mariam and Andreas needed to call in sick due to severe menstrual pain. Some participants worked even when they had severe menstrual pain, including Sofia, who experienced vomiting at work due to pain on more than one occasion:

*I have to be in*,* like*,* the foetal position. It’s hard to stand up straight. I’ve thrown up in the bathroom several times at work. I’m in so much pain that I have to sit down*,* like*,* I can’t work. But what can you do? You just have to grin and bear it. (Sofia*,* 27 years old)*

#### Lack of knowledge and understanding among cis men

The participants’ narratives reflected the menstrual stigma among cis men intertwined with the norms that menstruation is a feminine experience linked to cis girls and women. Several participants felt that people, particularly cis men, in their surroundings did not understand how menstruation and the menstrual cycle affected a menstruating person’s physical and mental health. For example, Noor experienced how men would minimise her pain: ‘*I’ve heard their opinions that it’s*,* like*,* it doesn’t hurt that much. You’re exaggerating. Like*,* they minimise it’.*

Most participants claimed that cis men lacked knowledge about menstruation, the menstrual cycle and menstrual hygiene products. This view was based partly on personal experiences and partly on general assumptions. Ester, who experienced severe mood changes, pointed out that not even her father knew anything about menstruation: ‘*I don’t think I could say that my dad would be able to explain what a menstrual cycle is or how it feels. For him*,* it’s like*,* a period*,* that’s just bleeding’.*

The participants found that cis men did not talk about menstruation or were uncomfortable doing so and that they viewed menstruation as disgusting. Some participants perceived that it was harder to share their experiences with people who had never menstruated. Andreas stressed that gender-neutral language should be used when talking about menstruation to include everyone, regardless of gender identity. The participants wished that everyone, regardless of gender, was taught about menstruation and the menstrual cycle. This could contribute to the normalisation of menstruation and increase the understanding of how menstruation and the menstrual cycle could affect one’s physical and mental well-being.

### Finding strategies for managing emotional discomfort and improving health

This theme describes how the participants perceived that their relationships were affected, how they took responsibility for the feelings of others and what strategies they used for managing symptoms and complaints related to the menstrual cycle.

#### Taking responsibility for others’ feelings

Caring about the people closest to them and feeling guilty were central in various contexts when the participants reflected on how their relationships with others were affected by their menstruation or menstrual cycle. Those who had a partner described that it was mainly this person who was affected by mood changes or PMS. Some talked with their partners to obtain emotional support or to explain their well-being or behaviour. This served as an advance warning to the partner.

*I usually say that I’m on my period and you can expect me to be slightly more irritable or upset*,* that little things I don’t usually react to might annoy me more or that kind of thing. (Farah*,* 26 years old)*

The symptoms described as PMS or mood changes could alter a participant’s way of thinking, feelings or reactions. Some reported that this affected their relationships with friends and partners, making them more offensive and easily angry. These altered thoughts or emotions could lead to doubts about their feelings for their partners and could result in a life crisis.

*I describe it as life feeling gray*,* but sometimes*,* it can actually be worse than that*,* you know. Sometimes you kind of end up in almost a life crisis*,* like*,* do I find my partner so annoying that it would be best to just leave him? And having to think of those thoughts once a month is probably more draining than I’d like to admit. (Freja*,* 28 years old)*

As a strategy to avoid hurting others, some participants reported avoiding meeting their friends. Some had trouble controlling their emotions but did not avoid meeting their friends because they needed social contact. It was clear that the participants found it difficult to control their emotions.

Several participants pointed out that emotions related to PMS, such as feeling sad, depressed, irritable or angry, led to unreasonable behaviours. Ester felt that it was hard for others to deal with her mood changes and that it was unsustainable to lose one’s temper once a month.

*It must be very hard from the outside to deal with someone who goes from one day not caring at all about stuff being left on the kitchen table to the next day thinking that person is crazy for not putting things away and then screaming and yelling*,* thinking that’ll make things better. It isn’t sustainable to lose control once a month*,* at least once a month*,* just because … because there are so many emotions involved. I have broken a broom*,* and this is not sustainable. (Ester*,* 25 years old)*

The feeling that their behaviour was not reasonable or sustainable also led to guilt and affected their well-being in general.

#### Strategies for managing emotional discomfort and symptoms

Self-care strategies to ease menstrual pain and emotional discomfort, such as PMS and mood changes, included pain relievers, heat packs, physical activity and masturbation.

*As there is quite a lot of frustration connected to my PMS*,* like anger*,* I exercise a lot. I often go for a run on*,* like*,* pure anger or anxiety. It’s not like exercise; it’s more like physical or being physically exhausted because then my entire system calms down. (Ester*,* 25 years old)*

Self-care, such as being kind to oneself and avoiding stressful situations, was also mentioned as a strategy for managing PMS and mood changes. Hanna described how she would watch a TV series, read a book or eat something tasty.

Talking about menstrual pain, PMS and mood changes with friends and family was another way for the participants to deal with symptoms. They sought support from someone close to them when things were difficult. Farah sought support from colleagues at work. As she worked in healthcare, she would usually tell her colleagues when she was menstruating and would ask for help when she felt that her condition affected patient contact. Andreas avoided discussing trans-related matters when he had PMS, as this made him feel worse.

Some participants sought healthcare, followed advice from healthcare professionals and started to use contraceptives to decrease complaints related to the menstrual cycle, with positive health outcomes. Julia and Robin, who experienced heavy bleeding and severe menstrual pain, were helped by contraceptives. For Farah and Ester, the contraceptive they were using helped alleviate their mood changes. By contrast, some participants had been in contact with healthcare for many years but did not receive treatment that could relieve their menstrual complaints. Denise, a regular client at the youth-friendly centre, received good reception and tried several treatments but did not obtain positive outcomes. She felt helpless and neglected, as the youth-friendly centre could no longer help her.

*There were two midwives*,* and I also met a doctor at the youth-friendly healthcare centre. They all said that we couldn’t do anything more*,* we couldn’t help you. It was awful. If not even a doctor or a midwife could help me*,* then how could I help myself? So I’ve been very sad about that and have felt neglected*,* helpless and hopeless. (Denise*,* 21 years old)*

Sofia, who had complaints about severe mood changes, had low confidence in healthcare, and the helplessness she felt was partly based on her friends’ experiences with healthcare contact. ‘*The people I know haven’t been helped with their menstruation issues. They may even experience a worse menstrual cycle than I do*,* so I’m like … okay*,* but if they can’t get help*,* then how am I supposed to get help’?*

Freja experienced healthcare-normalising complaints related to the female body. However, as the healthcare sector is under severe pressure, she thought that healthcare should not have to prioritise her menstrual-related complaints.

#### Menstruation as a sign of a healthy body and building a sense of community among cis women

When the participants were asked to reflect on their menstruation and whether there were any positive aspects, they presented a spectrum of views. Some participants who identified as cis women described menstruation as cool and powerful. Regardless of gender identity, other participants considered menstruation a sign of a healthy and functioning body.

*When it starts on time*,* I know that it’s a sign that I’m healthy and that everything is okay. It can be very soothing—with it being*,* like*,* cyclical and recurring … and that*,* like*,* my body is working the way it’s supposed to. (Hanna*,* 22 years old)*

Among those with regular monthly menstruation, missed or irregular menstruation was interpreted as having experienced a stressful lifestyle or situation. Furthermore, the participants perceived menstruation as a sign of maturity and associated it with being a woman and as something that contributed to a sense of community and sisterhood, which they described as positive. However, for trans men, the connection to womanhood created a sense of exclusion.

*If you think about the trans perspective*,* in some ways it excludes trans people from being able to share their experiences when you think that menstruation is something for women. It’s something that only women do*,* and it excludes trans men. (Robin*,* 25 years old)*

Andreas reflected on the norms related to menstruation and assumptions about who menstruates, stating that at his workplace, there was a ‘ladies’ box’ with menstrual pads in it, and that he changed the box name to a gender-neutral language.

## Discussion

The purpose of this study was to gain more knowledge about the experiences and perceptions of menstruation and the menstrual cycle among young adults in Sweden. Participants viewed menstruation as a sign of having a healthy and functioning body. Menstruation was associated with becoming a woman and fostered a sense of community and sisterhood, which cis women viewed as positive but for transmen, it often led to feelings of exclusion. Moreover, the results show that menstrual-related physical and psychological complaints limited the participants’ daily activities and social relationships, thus negatively affecting their sexual and mental health and well-being. Aside from managing physical and psychological complaints in public places during menstruation, they also had to deal with menstrual stigma and gender norms related to menstruation. Stigma and norms lead to adapted behaviours, such as avoiding participation in social activities or hesitating to ask for support. In addition, this study shows that access to accurate treatment that mitigates and decreases menstrual complaints limiting everyday life promotes menstrual health.

Our study results show that symptoms such as menstrual pain, heavy bleeding and PMS or mood changes limited the participants’ everyday life and social relationships, and that sexual and mental health were negatively affected. The level of reported menstrual complaints varied greatly between individuals, as did the effects on everyday life and general health. The participants’ descriptions of their menstrual-related symptoms were similar to those reported in previous studies [17, 31, 45]. However, those with both severe menstrual pain and heavy bleeding often felt limited in their everyday lives. This finding aligns with previous research indicating that menstrual pain and heavy bleeding are associated with a lower quality of life, resulting in absenteeism from school, work and social activities [31, 46, 47].

For the participants who identified as transgender men, the menstrual challenges they faced were not limited to menstrual complaints. As menstruation is rooted in society as a feminine normative experience and linked to cis women [48], the transmen described how menstruation contributed to their gender dysphoria and anxiety, consistent with previous research [17, 49]. To achieve menstrual health for all, inclusive menstrual health programmes are needed to support gender-diverse individuals, including transgender men and non-binary people [50]. Changing the language and narrative around menstruation is important to ensure an inclusive menstrual health programme.

Our findings suggest that the key barriers to menstrual health include stigma and norms related to menstruation. The normalisation of menstrual complaints also contributed to behavioural adaptations, such as delays in seeking healthcare, despite having symptoms that negatively affect their health. Some had experiences of not being taken seriously or not being prioritised when they sought care for complaints related to menstruation. This finding is in accordance with previous research showing that the normalisation of painful menstruation by both healthcare practitioners and families can contribute to late healthcare seeking despite the need, leading to delayed diagnosis of menstrual disorders [51–55]. As gender norms prescribe, having pain is constructed as normal for women. They are expected to carry the burden of it.

However, this study also reveals the opposite experience, with some participants receiving early and appropriate treatment for symptoms, such as heavy bleeding, severe menstrual pain and PMS, resulting in the relief of symptoms and improved quality of life. This finding indicates that access to adequate treatment that mitigates and decreases symptoms limiting everyday life is an important factor in promoting menstrual health [3]. Gambadaduari et al. suggest that education, screening, and clinical competence are important tools to reduce the burden of menstrual symptoms during adolescence and to prevent long-term consequences [30].

Moreover, consistent with earlier studies [17, 56, 57], the participants in this study used various strategies and behavioural adaptations in their daily lives to manage menstruation and its associated symptoms. The participants’ behavioural adaptations related to menstrual stigmatisation and gender norms also reflected internalised menstrual stigma, aligning with findings from previous studies [17, 58]. Previous research suggests that women with PMS are portrayed as overly sensitive and emotional, which is viewed as negative and undesirable behaviour [38]. This view was embodied by the participants in this study who experienced PMS. The participants in this study stayed away from family and friends or tried to avoid stress and conflicts to avoid hurting people close to them, as revealed in a previous study [59]. Furthermore, they described their behaviour during PMS as inappropriate and dictating how women should behave, thus affecting their self-perception. Participants took responsibility for the other’s feelings, adapting their own behaviour —particularly their anger— to avoid upsetting others’ feelings, in accordance with societal expectations that discourage women from displaying aggression [33]. Although, PMS, as a culturally constructed symbol, also provided a justification for expressing their anger.

The participants experienced being misunderstood and minimised by cis men regarding menstrual-related complaints, which highlights a non-supportive environment for menstruating people. Previous studies have shown that cis men lack knowledge of menstrual health and understanding of menstruating people [60, 61]. Future research should identify how cis men, such as fathers, brothers, male colleagues and peers, could contribute to a supportive environment for menstruating people. An open discussion on stigma and norms regarding appropriate behaviour could also be productive.

Furthermore, our study reveals that while managing their emotional discomfort, pain, severe bleeding, and menstrual management in public places during menstruation, the participants also dealt with menstrual stigma and gender norms related to menstruation. Previous research has shown that the sociocultural context of stigmatisation and gender norms could manifest in strict expectations of people who menstruate and expectations that are coercive and internalised, negatively affecting mental health [17]. This led to decreased participation in activities [17], which was also observed in our findings. Previous studies have also shown that refraining from social activities is related to the fear of blood leakage and the disclosure of menstruation [17, 28]. The participants in this study described similar experiences, indicating that they were affected by menstrual stigma. Johnston-Robledo and Chrisler argue that menstrual stigma negatively affects girls’ and women’s self-esteem, body image and self-presentation [36]. According to McHugh, ‘menstrual moaning’, may negatively influence women’s attitudes toward menstruation and contribute to the perpetuation of menstrual shame [39].

The participants described their sexual desires as changing depending on where they were in their menstrual cycle. For example, increased sexual desire during menstruation was mentioned, with some participants considering it to be partially positive. Norms about not having sex during menstruation were among the reasons why some abstained, which is consistent with other studies [62–64]. A study by Fahs reveals that positive experiences of sex during menstruation were more common among white, lesbian and bisexual women than among heterosexual women of colour [64]. Not having sex during menstruation could be due to decreased sexual desire, as some people feel low, bloated or tender. Consistent with previous research [65–68], our study reveals that symptoms such as severe menstrual pain and heavy bleeding had a negative effect on sexuality.

### Implications of the findings

The results indicate that menstrual health can be improved by access to adequate treatment that alleviates symptoms that limit daily activities and quality of life. Hence, to promote menstrual health of menstruating young adults, organised and systematic screening of menstrual cycle-related symptoms should be provided within student health services at schools and universities, and primary healthcare. Moreover, when individuals seek help for menstrual cycle-related complaints, healthcare providers need to be responsive to their needs in relation to menstrual complaints.

Our results also show that stigma and lack of understanding limited the lives of menstruating people, not only when they menstruated but also due to the physical and psychological complaints they experienced during the premenstrual phase. The experiences and interpretations of menstruation are clearly biological as well as cultural, and attention needs to be paid to all aspects of menstruation to improve overall menstrual health. Knowledge-increasing efforts among cis men and non-menstruating people can contribute to a supportive environment in which menstruating people are met with respect instead of condescending attitudes. Furthermore, policymakers should consider integrating stigma-reducing efforts to improve general awareness and to accelerate the achievement of gender equality.

### Strengths and limitations

One of the strengths of this study is that the analysis process involved a multidisciplinary team consisting of five researchers with different preunderstandings, competences and professions. This triangulation of different professional perspectives (midwife and reproductive health expert, public health expert, gender specialist) made it possible to view the data from several angles, which enriched the analysis and interpretation of the data. The triangulation and use of quotations increased the credibility and transparency [69, 70]. As for the reflexivity, we acknowledged that our characteristics, preunderstandings and prior knowledge, as well as own personal biases and social positions, might have influenced the interpretation of the data and discussion of findings. Reflexivity also involves being critically self-aware of one’s role in the processes of data collection. For example, one of the researchers was recognised by one of the participants during the interview, which could have affected the answers in that case. As for dependability, we strive to provide a transparent account of the study process, from the data collection to the analysis and reporting of the findings. In terms of limitations, several possible biases arose due to various types of missing which might affect transferability —the extent to which results can be applied to other groups or contexts [70]. Despite our efforts to recruit participants with diverse backgrounds in terms of LGBTQ people, country of birth, language, and migration experiences, we were unable to fully achieve this goal. Regarding language, the information letter was written in Swedish, meaning that individuals who did not speak Swedish were unable to access the information and were thus indirectly excluded from the study. Certain groups or perspectives may be underrepresented, limiting the transferability of study findings to specific subgroups. The lack of diversity might result in missing critical insights or experiences related to the research questions, especially those tied to cultural or linguistic factors. It is possible that other experiences of menstrual health would have been revealed if more foreign-born and LGBTQ people had been interviewed including people with disabilities.

To minimize potential bias in experiences of menstrual complaints and diagnoses, we chose not to recruit participants through health services. Despite this, our results revealed that menstrual complaints were widespread among participants, which may indicate an overrepresentation of such issues. However, the widespread experiences of menstrual complaints among participants align with robust evidence confirming that such occurrences are common among young people [71]. The overrepresentation of menstrual issues could be explained by the fact that individuals who had experienced or were currently experiencing issues were particularly motivated to participate in the interview and share their experiences with us. The range of education background, occupation and gender identity among the participants resulted in both similarities and disparities in the results, which were based on the Swedish context. However, the results were similar to the experiences and perceptions of menstrual health seen among menstruating people in other countries. In particular, the experiences of menstrual stigma did not appear to be tied to any specific context or country, although they could take on different forms.

## Conclusion

The findings indicate that stigma and norms related to menstruation create challenging situations limiting menstruating people’s everyday lives, social participation, and reluctance to seek healthcare despite needing to. To promote the menstrual health and well-being of menstruating young adults in Sweden, organised and systematic screening of menstrual cycle-related symptoms should be provided within student health services at schools and universities, and primary healthcare. Policymakers should consider integrating stigma-reducing efforts into public health interventions to improve general awareness and advance gender equality.

## Electronic supplementary material

Below is the link to the electronic supplementary material.

Supplementary Material 1

Supplementary Material 2

## Data Availability

The datasets generated and analysed during the current study are not publicly available due to the data containing information that could compromise research participant privacy/consent, but are available from the corresponding author (eva.akerman.2@ki.se) on reasonable request.

## Abbreviations

LGBTQ: Lesbian, gay, bisexual, transgender and queer
PMS: Premenstrual syndrome
