# Publisher Correction: Navigating menstrual stigma and norms: a qualitative study on young people’s menstrual experiences and strategies for improving menstrual health

**DOI:** 10.1186/s12889-025-22328-9

**Published:** 2025-03-31

**Authors:** Eva Åkerman, Anna Wängborg, Maria Persson, Renita Sörensdotter, Marie Klingberg-Allvin

**Affiliations:** 1 Department of Women’s and Children’s Health, Karolinska Institutet, Tomtebodavägen 18A, Stockholm, 171 77 Sweden; 2 Department of Public Health Sciences, Stockholm University, Albanovägen 12, Stockholm, 106 91 Sweden; 3 Centre for Gender Research, Uppsala University, Villavägen 6, Uppsala, 752 36 Sweden

**BMC Public Health (2024) 24:3401**

10.1186/s12889-024-20936-5

The original publication of this article contained several citation & reference errors due to the publication process. All references after 40 were incorrectly ordered. No references or citations have been removed/added. The original publication has been corrected. The publisher apologizes to the authors & readers for the inconvenience caused.

