# Supplementary material for: Navigating menstrual stigma and norms: a qualitative study on young people’s menstrual experiences and strategies for improving menstrual health
Source: BMC Public Health. 2024 Dec 17;24:3401. doi: 10.1186/s12889-024-20936-5 (PMC11654398; doi:10.1186/s12889-024-20936-5)
Supplement: Supplementary file 1 — Supplementary Material 1 [file 12889_2024_20936_MOESM1_ESM.docx]

**Supplementary material**

**Interview guide: young people’s experiences of menstrual health**

**Icebreaker**

- What is the first thing that comes to your mind when I say “period”?
- Are there any other words/terms you use when talking about periods with your friends? What do they mean?
- What do you think of when I say “menstrual cycle”?

**Experiences of menarche**

- How old were you when you got your first period? Did you know what to do?
- Did you have access to menstrual products during your first period? How? If no: what did you do to access menstrual products?
- Do you remember how you felt when you got your first period?
- Ask follow-up questions about both positive and negative experiences (both emotional and physical).

**Support during adolescence**

- Who did you talk to when you got your first period?
- Do you remember what you talked about? What?
- How did this person react when you told them about your first period?
- If you needed advice about periods, who did you talk to when you were younger?
- Could you talk openly about periods with your family? Friends?
- Looking back, who do you consider having been the most important source of information about periods when you were younger, after you got your first period?
- Who did you talk to about menstrual products? Who bought menstrual products for you when you were younger?
- Looking back, who do you consider having been the most important source of information about menstrual products? Any other sources?

**Knowledge about menstruation and the menstrual cycle**

- What did you know about periods before you got your first period? From whom/where did you get the information?
- What did you know about menstrual products before you got your first period? From whom/where did you get the information?
- What was your sex education like in elementary school? In which subject was sex education included?
- What did you learn about periods? What did you learn about the menstrual cycle? What was missing?
- Looking back, is there anything you wish you had known about periods before you got your first period? About the menstrual cycle? About menstrual products?

**Experiences of menstruation, menstrual cycle, and menstrual products**

- How many days do you usually have your period? Probes: type of menstrual product, and frequency of changing menstrual product.
- How often do you have your period (referring to the menstrual cycle)?
- Do you experience regular periods? Probes: Do you know when to expect your next period?
- You mentioned using xxx (menstrual product). Why do you use this product? What do you think about the price of xxx (menstrual product)?
- Do you use any contraceptives? If yes: What type of contraceptive?
- What is the main reason for using contraceptives?

**Impact of menstruation/menstrual cycel on health and well-being, self-care and strategies**

- How do you perceive your health when you have your period?
- Can you describe how a typical menstrual cycle looks like for you? What complaints/symptoms occurs and when? Clarify if needed: Is there a difference in how you feel before, during, and/or after your menstrual bleeding?
- How does your menstrual cycle-related symptoms affect your health?
- When in your menstrual cycle to you feel the best?
- When in your menstrual cycle to you feel the worst?
- If having complaints: Do you take any medications (pain relievers, etc.)? Do you have any diagnoses?
- What do you do to alleviate the complaints/symptoms (strategies/self-care)?
- If no diagnosis: Have you sought medical care? Do you talk to anyone about your complaints/symptoms? Who?

Follow-up questions for those who have sought care:

- - Would you like to elaborate, where did you seek care?
  - How did you experience the treatment from the healthcare professional?
  - If positive experiences, what was good?
  - If negative experiences, what was not good? What support did you wish for/hope for? Did you receive help for what you sought care for?

**In-depth questions**

*Skip the questions below if the participant has shared these experiences earlier.*

**Impact of menstruation/menstrual cycle on mental health**

- When you have your period, how does it affect your mental health? Any differences regarding your mental during other phases of the menstrual cycle, positive/negative impact? Would you like to elaborate?
- What do you do to alleviate the complaints/symptoms (strategies/self-care)? Is there anything you avoid doing?
- Do you talk to anyone about your symptoms? Who?

**Impact of menstruation/menstrual cycle on physical health (body and functions)**

- When you have your period, how does it affect your physical health (body and functions)? Any discomfort/pain/heavy bleeding? Any differences regarding your physical health during other phases of the menstrual cycle, positive/negative impact? Would you like to elaborate?
- What do you do to alleviate the complaints/symptoms (strategies/self-care)? Is there anything you avoid doing?
- Do you talk to anyone about your symptoms? Who?

**Impact of menstruation/menstrual cycle on daily life, education, work, and lifestyle**

- When you have your period, how does it affect your daily life? Education/work? Leisure activities? Social activities? Lifestyle/habits: physical activities, sleep, and food?
- Is there anything you particularly avoid in your daily life when you have your period? Why? Anything you particularly do different when you have your period?
- Would you like to elaborate?
- Any differences regarding your daily life during other phases of the menstrual cycle, positive/negative impact? Education/work? Leisure activities? Social activities? Lifestyle/habits: physical activities, sleep, and food? If yes: is there anything you particularly avoid in your daily life when you experience these complaints? Anything you particularly do different? Would you like to elaborate?

**Impact of menstruation/menstrual cycle on relationships and sexual health**

- When you have your period, how does it affect your relationships with your family, friends, colleagues, and (your partner)? Any differences during other phases of the menstrual cycle, positive/negative impact? Would you like to elaborate?
- When you have your period, how does it affect your sexual desire? Any differences regarding your sexual desire during other phases of the menstrual cycle, positive/negative impact? Would you like to elaborate?

**Menstrual health literacy**

- What are your thoughts when your period is delayed? If it is irregular? If you bleed differently than usual?
- Besides your own menstrual cycle-related experiences, what other symptoms/complaints have you heard of?
- When was the last time you sought information about menstruation? About the menstrual cycle?
- What information did you seek? What was the reason for seeking the information? How do you judge if the information you found is reliable?
- How did you learn what you know about menstruation today? About menstrual cycle? Is there anything you would like to learn more about? What?
- If you were to experience complaints related to your period, where would you turn for support? For menstrual cycle complaints?

**Thoughts and attitudes towards menstruation/menstrual cycle**

- How do you think your experience of menstruation has changed over time, from your early years to how you experience it today?
- Is your experience of menstruation something you share with others currently? If so, with whom? Probe: partner, parent, sibling, friends, colleagues…
- When was the last time you talked about your period with someone? About your menstrual cycle?
- What do you think others around you think about menstruation today? About the menstrual cycle? Probe: partner, parents, siblings, friends, colleagues at work/school?
- Do you feel that there are differences in thoughts about menstruation based on gender? Would you like to give examples?

Questions for foreign-born participants/participants with foreign-born parent:

- In your home country where you were born, how do people talk about menstruation?
- Are there any restrictions during menstruation? If yes: would you like to give examples?
- Are there recommendations/self-care strategies for what you should do when you have your period? If yes: would you like to give examples?
- Are there any myths about menstruation? If yes: would you like to elaborate?

**Other**

- Ask about positive aspects of menstruation if participants haven’t been mentioned any earlier.
- What would your ideal period look like?
- What would your ideal menstrual cycle look like?
- Is there anything I have forgotten to ask or anything you would like to add?
